# Efficacy of Dr. SKS Hair Booster Serum in the Treatment of Female Pattern Alopecia in Patients With PCOS: An Open-Label, Non-randomized, Prospective Study

**DOI:** 10.7759/cureus.44941

**Published:** 2023-09-09

**Authors:** Stuti Khare

**Affiliations:** 1 Dermatology, Elements of Aesthetics, Mumbai, IND

**Keywords:** female pattern alopecia, treatment, polycystic ovary syndrome, hair loss, androgenetic alopecia

## Abstract

Background

Many patients with polycystic ovary syndrome (PCOS) exhibit female pattern hair loss (FPHL). A more advanced, efficient, and suitable therapeutic approach is required to effectively manage FPHL in patients with PCOS.

Aim

Dr. SKS Hair Booster Serum is composed of copper, niacinamide, hyaluronic acid, thiamine, riboflavin, and biotin; each of these constituents has demonstrated individual efficacy in promoting hair growth and enhancing hair quality. We hereby assess the effectiveness of this novel hair formulation in treating FPHL in PCOS.

Methods

This was an open-label, non-randomized, multicenter, prospective, large study with a wide range of age groups. The study involving 1,000 females aged 25-50 years, diagnosed with PCOS and having complaints of FPHL with Ludwig grades I and II. Each patient received a monthly session of Dr. SKS Hair Booster Serum, with 1 mL of serum administered through injection into the superficial layer (dermis) of the scalp using a tiny infusion via an insulin syringe, mesotherapy, or via a derma roller/derma pen. All the patients were subjected to standard global photography, video microscopic assessment (vellus hair counts, terminal hair counts, and hair shaft diameter), and a subject self-assessment questionnaire at baseline and six months after the treatment.

Results

After six months of the treatment, the hair shaft diameter, terminal hair counts, and hair growth rate were significantly increased than baseline (p≤0.0001), and a significant reduction was noted in vellus hair counts than baseline measurement (p<0.00001). These findings are suggestive of improved hair regrowth after the treatment. No adverse events were recorded during the study. Statistically significant improvements were observed in hair parameters (overall hair fall rate, hair texture, hair volume, and scalp itching) after six months of treatment than baseline.

Conclusion

Dr. SKS Hair Booster Serum has been shown to be an effective treatment for FPHL in patients with PCOS. This study marks the first investigation into the use of Dr. SKS Hair Booster Serum in patients with PCOS.

## Introduction

Polycystic ovarian syndrome (PCOS) is a prevalent hormonal and endocrine condition that primarily affects women after puberty. It is marked by polycystic ovaries, infertility, oligo- and/or anovulation, and hyperandrogenism [1]. PCOS affects 5% to 18% females worldwide [2]. Around 20% to 30% of patients with PCOS exhibit female pattern hair loss (FPHL) or female pattern alopecia - a typical form of androgenetic alopecia in females and hormonally marked by directed diffuse hair loss on the scalp [3]. PCOS typically causes vertex thinning while maintaining the frontal hairline, but, in rare cases, it can cause central scalp hair loss similar to androgenetic alopecia. The effects of hyperandrogenism include an increase in 5-alpha reductase and androgen receptors, and a decrease in cytochrome p450 enzyme levels. These changes lead to a short anagen phase, miniaturization of terminal hairs, and eventually the conversion to vellus hair [4]. The prevalence of FPHL increases from 12% to 50% with the increasing age from 20-80 years [5]. Compared to males, most of the females due to FPHL suffer from adverse psychosocial consequences such as anxiety disorder, social phobia, depression, or paranoid disorder, and have a poor quality of life [4,6].

Despite the fact that the management of FPHL with topical minoxidil [7,8], dutasteride and finasteride [9-13], flutamide [14,15], cyproterone acetate [16], and oral and topical spironolactone [17] has widely been discussed in the literature, there is an increasing need and scope for a better, more effective, and more appropriate therapeutic approach for the management of FPHL in patients with comorbid PCOS due to the complexity, multiple pathomechanisms, widespread impact on patients, and negative impact on quality of life. Against this background, we examined the effectiveness of Dr. SKS Hair Booster Serum in treating FPHL in patients with PCOS.

## Materials and methods

Study participants

It was an open-label, non-randomized, multicenter, prospective study conducted from September 2021 to October 2022. Female participants were included if they met the following criteria: a) diagnosed with PCOS according to hormonal assessment and sonography, b) having FPHL (categorized as Ludwig's hair loss grades I and II) based on clinical examination, c) meeting at least two of the three broader Rotterdam's criteria of PCOS, i) ovulatory dysfunction, ii) polycystic ovarian morphology, and iii) hyperandrogenism, and d) not responding for a period of a year or longer to a treatment regimen that included topical minoxidil 5% and oral finasteride/spironolactone/contraceptives.

Exclusion criteria were as follows: a) experiencing hair loss (less than 6 months), b) patients having hematologic and/or autoimmune diseases, seborrheic dermatitis, known medication allergies, other scalp skin diseases, or suspected or proven malignancies, c) pregnant women and nursing mother, and d) all patients following their food and exercise regimens to treat PCOS. Patients were excluded based on the observation findings and the assessment of patient history.

Evaluation of female pattern hair loss

FPHL is assessed based on following three hair patterns by using different scales: a) diffuse thinning on the crown area with preservation of the hairline in frontal region (5-point Sinclair scale and 3-point Ludwig scale), b) characteristic Christmas tree pattern, which includes widening and thinning of the scalp's central region and a breaching of the hairline in frontal region (Olsen scale), and c) hair thinning related to bitemporal recession (Hamilton-Norwood scale).

Injections of the formulation to the scalp

Dr. SKS Hair Booster Serum is formulated with a distinctive blend of chemical components including copper (10 mg/L), niacinamide (5 mg/L), hyaluronic acid (0.25 mg/L), thiamine (0.2 mg/L), riboflavin (0.2 mg/L), biotin (0.025 mg/L), and water (aqua) as required. A total of 1 mL of Dr. SKS Hair Booster Serum injection was administered into the superficial layer (dermis) of the scalp with tiny infusion via an insulin syringe, mesotherapy, or a derma roller/derma pen once a month up to six months. Initial changes and observable outcomes were noticeable from the second to third month following the treatment. Efficacy and safety outcomes were evaluated at baseline and six months following the treatment.

Scalp assessment and evaluation

Global Photographic Assessment

Standardized clinical images of the head were taken for clinical evaluation at baseline and six months following treatment. Images of the vertex and superior frontal areas of the scalp were taken with the standardized method. Each image was examined by two independent dermatologists (range: 0-10; 0=growth, and 10=full, thick hair growth). Next, scores were calculated as an average and compared between baseline and six months following the treatment.

Video Microscopic Examination

Video microscopic digital handheld camera was used to take images. Each image was taken with a fixed position at the center of the scalp (20 cm behind or posterior to the glabella). Hair count/cm^2^, shaft diameter (µm), vellus hair counts (cm^2^), and terminal hair counts (cm^2^) were also assessed with images and specialized software TrichoScan® Professional version 3.7.27.124 (Tricholog GmbH and Datinf GmbH, Freiburg, Germany).

Subject Self-Assessment Questionnaire

Patients were asked to rate the questions in terms of the efficacy of the treatment (scalp itching, hair texture, hair volume, and hair fall rate) following six months of the treatment than baseline on a 5-point scale (range: 0-5) (Table 1).

**Table 1:** Grading of the hair parameters

Parameters	Grading
Hair fall rate	1 = ≥100 (worst possible situation)
	2 = 50–100
	3 = 30–50
	4 = 10–30
	5 = ≤10 (best possible situation)
Hair texture	1 = Rough and frizzy (worst possible situation)
	2 = Rough
	3 = Normal
	4 = Soft
	5 = very soft (best possible situation)
Hair volume score	1 = Very less volume (worst possible situation)
	2 = Less volume
	3 = Average
	4 = Good volume
	5 = Excellent (best possible situation)
Grading hair fall	0 = No itching (best possible situation)
	1 = Negligible itching-no concern
	2 = Mild itching
	3 = Moderate itching
	4 = Severe itching
	5 = Very severe itching-compromises the daily routine

Safety assessment: Adverse events were noted throughout the duration of the study.

Statistical analysis

Descriptive data are expressed as mean and standard deviation. Categorical data are expressed as number and percentage. Comparison of efficacy parameters was done using a parametric test (i.e. paired t-test) between baseline and six months after the treatment. A p-value of <0.05 was considered statistically significant. The Statistical Package for the Social Sciences (SPSS) Version 22 (IBM Corp., Armonk, NY) was used for all statistical analyses.

## Results

A total of 1,000 patients with female pattern alopecia in PCOS, with a mean age of 38.43 ± 2.03, were assessed. There were 730 (73%) females with grade I and 270 (27%) females with grade II Ludwig scale.

Global photographic assessment

**Figure 1:**
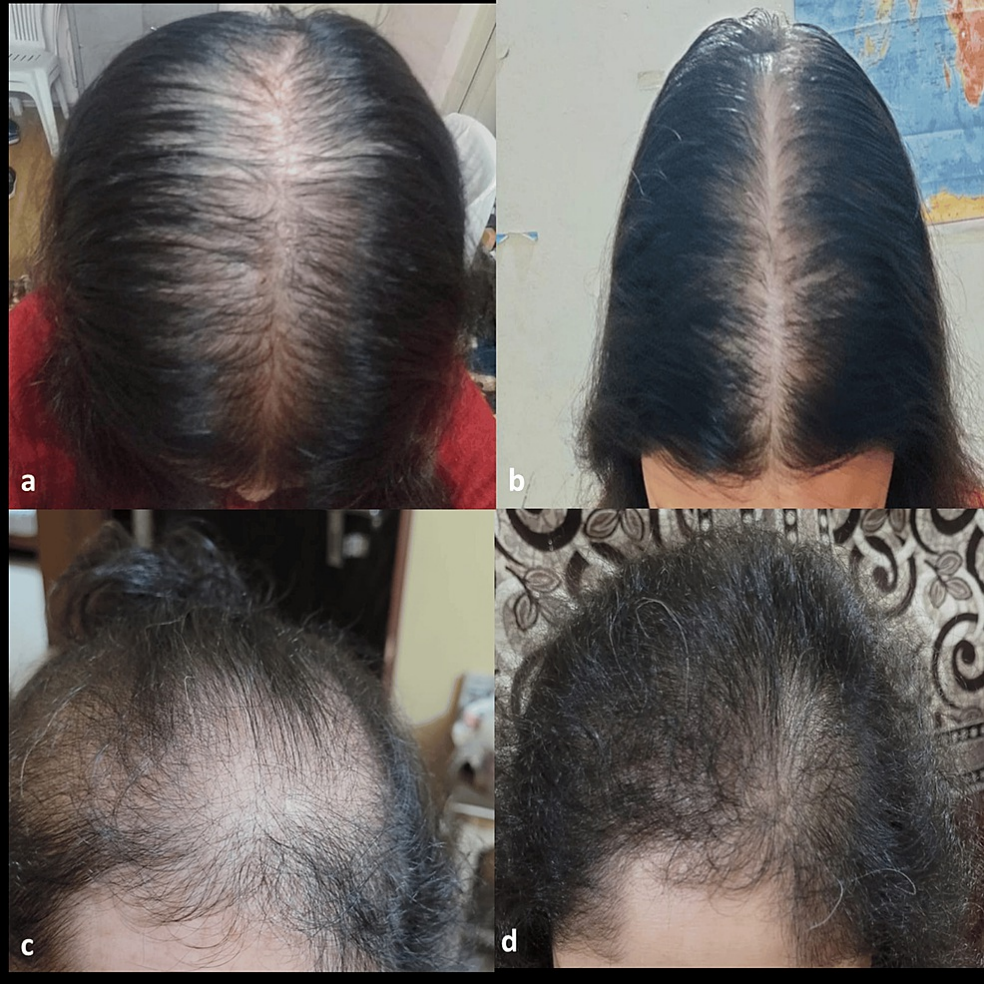
Significant hair growth in female pattern androgenetic alopecia six months after the treatment (b, d) compared to baseline (a, c).

As depicted in Table 2, the mean global photographic assessment (GPA) score was significantly (p<0.00001) improved from 2 to 9.95 at six months following the treatment than baseline, leading to a 79.8% change, indicating satisfactory hair growth (Figure 1).

**Table 2:** Global photographic assessment score at baseline and six months after the treatment

Reviewer	At baseline	After six months
Reviewer 1	2	9
Reviewer 2	2	9.5
Mean	2	9.25

Video microscopic examination

Terminal hair counts significantly increased from 77.33 to 91.33 six months after the treatment compared to baseline, indicating a 13.51% change. A significant increase (27.67 to 32.00; 13.50% change) in hair shaft diameter was observed six months after the treatment compared to baseline. There was a percentage reduction of 35.6% (51.80 to 38.20) in vellus hair counts at six months after the treatment compared to baseline (Table 3). All these finding reflected better hair regrowth.

**Table 3:** Mean score of vellus hair counts, terminal hair counts, and hair shaft diameters at baseline and six months after the treatment Δ indicates changes in values from baseline to after six months.

	Mean at baseline	Mean after six months	Δ	P-value
Terminal hair counts (cm^2^) at 20 cm	77.33	91.33	14	<0.00001
Vellus hair counts (cm^2^) at 20 cm	51.80	38.20	-13.60	<0.00001
Hair shaft diameter at 20 cm (µm)	27.67	32.00	3.25	0.0001

Subject self-assessment questionnaire 

Statistically significant improvements were observed in overall scalp itching, hair texture, hair fall rate, and hair volume following six months of treatment than baseline. At the end of the treatment, no itching was reported, and with the consistent use of the test product, hair fall completely ceased (Table 4).

**Table 4:** Changes in self-assessment questionnaire

Parameters	Mean at baseline	Mean after six months	P-value
Hair fall rate	1.5	4.8	0.00003
Hair texture	2.3	4.7	0.00002
Hair volume score	1.8	4.3	0.00028
Grading hair fall	1.3	0	-
Scalp itching	0.7	0	-

Safety assessment

Skin intolerance was not observed during the study. Dr. SKS Hair Booster Serum was deemed safe for dermatological use.

## Discussion

The current study demonstrates that our hair serum formulation enhanced hair growth rate, vellus hair counts, and hair shaft diameter in FPHL in patients with PCOS. The main findings that emerged after six months of the treatment are as follows: 1) significantly improved mean GPA score, leading to a 79.8% change, indicating satisfactory hair growth, 2) significantly improved terminal hair counts and hair shaft diameter after the end of the treatment from baseline, 3) significantly reduced vellus hair counts, representing a 35.6% change, and 4) significantly improved overall hair texture, scalp itching, hair volume, and hair fall rate.

Dr. SKS Hair Booster Serum has received FDA approval and provisional patent approval from the Indian Patent Office. Through an open-label, non-randomized, multicenter, prospective study of 1,000 patients, Dr. SKS Hair Booster Serum has already proved its safety and efficacy in the treatment of androgenetic alopecia in both males and females with 95% patient self-assessment score [17]. We hereby sought to evaluate the efficacy and safety of this serum in the treatment of FPHL in patients with PCOS.

It contains a blend of micronutrients and multivitamins, including copper, niacinamide, hyaluronic acid, thiamine, riboflavin, and biotin. Copper increases hair follicle size, resulting in thicker hair [18,19]. Hair thickness clearly improves with niacinamide [20,21]. In addition to smoothing the cuticles and strengthening the hair strands by lowering the chance of hair breakage, hyaluronic acid also stores moisture [22,23]. Thiamine, or vitamin B1, expands hair follicles and causes them to produce thicker hair [24]. Biotin promotes healthy hair growth [25-27], and vitamin B2 (riboflavin) creates the cellular energy needed by hair follicles [28,29]. Amalgamation of all these ingredients aid in promoting hair regrowth and hair regeneration.

In literature, topical minoxidil [7,8], dutasteride and finasteride [3,9-12], flutamide [13,14], cyproterone acetate [15], and oral and topical spironolactone [16] have been proved to be fruitful in treating FPHL associated PCOS. However, these treatments are associated with several side effects. As such, Lucky et al. [7] and Olsen et al. [8] discovered that 5% topical minoxidil was more effective than 2% topical minoxidil for treating FPHL. However, these were associated with an increased occurrence of pruritus, local irritation, and hypertrichosis. In a retrospective study of 30 women, Boersma et al. [9] found similar efficacy of finasteride (1.25 mg) and 3 years of treatment of dutasteride (0.15 mg) in improving hair density and thickness, but found more efficacy with three years of treatment of dutasteride (0.15 mg) in women's central and vertex sites. Several investigations found finasteride at dosages of 2.5 mg and 5 mg to improve FPHL in postmenopausal normoandrogenic women [3,10, 11]. In a multicenter, double-blind, randomized controlled trial involving 137 postmenopausal women with mild-to-moderate FPHL, Price et al. [12] found no significant differences in changes of hair loss by hair count between finasteride 1 mg and placebo after one year of treatment. However, finasteride and dutasteride are associated with drug-related adverse events (folliculitis), headache, nausea, hot flashes, and depression. In 36 hyperandrogenic young women with FPHL participating in a randomized, open-label trial, Carmina et al. [13] found that 250 mg/day of flutamide significantly decreased Ludwig scores (21%).

Paradisi et al. [14] in a prospective non-blinded cohort study found a significant decrease in alopecia scores following 12 months of flutamide therapy. Side effects observed with the use of flutamide are headache, respiratory tract disorders, nausea and/or vomiting, diarrhea, dry skin, and reduction of libido. Vexiau et al. [15] reported that high dosages of cyproterone acetate (50 to 100 mg/day) were beneficial in treating hyperandrogenic women with FPHL. In a systematic analysis, Wang et al. [16] demonstrated the effectiveness of oral and topical spironolactone in treating alopecia. Topical treatment was more effective when used in conjunction with other treatments such as oral or topical minoxidil than when used alone. Topical spironolactone is associated with contact dermatitis, mainly presented as pruritus, burning and scaling, whereas oral spironolactone is associated with dizziness/headache, menstrual disorder, rash, nausea, increased urination, breast tenderness, hyperkalemia, facial hypertrichosis, aggravation of trichomoniasis, scalp pruritus, increased scurf, edema of the limbs, palpitation, and postural hypotension. Our product shows good efficacy in terms of hair growth rate, vellus hair counts, and hair shaft diameter in FPHL in PCOS, with no side effects.

The current study found a significantly improved mean GPA score from 2 to 9.95 at six months after the treatment than baseline. GPA score showed marked improvement over six months. We have found significant improvement in terminal hair counts and hair shaft diameter, as well as reduction in vellus hair counts after six months of the treatment, reflecting excellent hair regrowth in PCOS patients. Vellus hair is very short, fine, and usually non-pigmented, whereas terminal hair is longer, thick, and pigmented. Excessive amounts of androgen hormones in the female system have an ability to transform vellus hairs into terminal hairs.

There are certain limitations that should be acknowledged in the present study. Firstly, hair serum product has not been compared to the currently available therapy options; in this regard, we recommend randomized studies with an active comparator and long-term follow-up. Moreover, mechanisms through which Dr. SKS Hair Booster Serum encourages hair development should also be investigated, particularly considering hormonal imbalances in PCOS that impact the hair follicles and lead to female pattern alopecia.

## Conclusions

Dr. SKS Hair Booster Serum has been shown to be an effective treatment for FPHL in PCOS. In PCOS individuals with FPHL, it promotes hair growth, increases hair density, and lessens hair loss. With no adverse effects and good tolerance, it is a well-tolerated alternative for FPHL in patients with PCOS.
